# Into the dark: patterns of middle ear adaptations in subterranean eulipotyphlan mammals

**DOI:** 10.1098/rsos.170608

**Published:** 2017-09-20

**Authors:** Daisuke Koyabu, Misato Hosojima, Hideki Endo

**Affiliations:** The University Museum, The University of Tokyo, Hongo 7-3-1, Bunkyo-ku, 113-0033 Tokyo, Japan

**Keywords:** fossoriality, moles, geometric morphometrics, impedance matching, low-frequency sound

## Abstract

Evolution of the middle ear ossicles was a key innovation for mammals, enhancing the transmission of airborne sound. Radiation into various habitats from a terrestrial environment resulted in diversification of the auditory mechanisms among mammals. However, due to the paucity of phylogenetically controlled investigations, how middle ear traits have diversified with functional specialization remains unclear. In order to identify the respective patterns for various lifestyles and to gain insights into fossil forms, we employed a high-resolution tomography technique and compared the middle ear morphology of eulipotyphlan species (moles, shrews and hedgehogs), a group that has radiated into various environments, such as terrestrial, aquatic and subterranean habitats. Three-dimensional geometric morphometric analysis was conducted within a phylogenetically controlled framework. Quantitative shapes were found to strongly reflect the degree of subterranean lifestyle and weakly involve phylogeny. Our analyses demonstrate that subterranean adaptation should include a relatively shorter anterior process of the malleus, an enlarged incus, an enlarged stapes footplate and a reduction of the orbicular apophysis. These traits arguably allow improving low-frequency sound transmission at low frequencies and inhibiting the low-frequency noise which disturbs the subterranean animals in hearing airborne sounds.

## 1. Introduction

The auditory processing system of terrestrial mammals is mostly tuned to hear sonic range (below 20 Hz) and/or high-frequency sounds (above 20 kHz) which are transmitted through air [1,2]. The middle ear ossicles, which are key innovations for mammals [3], play an essential role in transmission and impedance transform of airborne sound [4]. Some mammals that secondarily shifted their habitat from above-ground environments underwent evolutionary changes of their auditory mechanisms, as both the media propagating sound as well as the prevalent frequency of sound differ from the terrestrial environment [5–9]. In particular, improvement of non-visual perception is critical for living in darkness where available light is limited [10]. As low-frequency sound is better propagated than high-frequency sound through the dense medium of soil [11], subterranean mammals are known to depend more on low-frequency sound [5,12,13]. Previous studies have identified that subterranean mammals exhibit larger tympanic membranes and stapes footplates, loose connections between the malleus and tympanic bones and reduced middle ear muscles [2,12,14–21]. However, previous functional studies have rarely considered the phylogenetic effect on middle ear morphology due to a lack of rigorous phylogenetic frameworks, and even distantly related taxa have often been compared. Morphology can be highly biased by phylogenetic history and may not necessarily reflect functional specialization [22]. Phylogenetically controlled investigations are still lacking, and it remains unclear how morphological traits of the middle ear ossicles may have evolved with functional specialization.

Here, in order to identify the respective patterns for terrestrial, subterranean and aquatic lifestyles, we compare the middle ear morphology of 30 eulipotyphlan species, including shrews, hedgehogs and talpids. We clarify the morphological characteristics specific to each lifestyle using three-dimensional geometric morphometric analysis and impedance transform analysis within a phylogenetically controlled framework. Recently, a number of geometric morphometric analyses of the inner ear revealed that its morphology can predict modes of locomotion and agility [23–30]. However, to our knowledge no study has yet attempted to apply geometric morphometric approaches to the middle ear in mammals, except for hominids [31,32]. This is because the middle ear ossicles are often lost or do not preserve their original orientation in dried skulls, therefore it is necessary to collect wet specimens to study the ossicles. By contrast, the bony labyrinth, housed in the cochlea, is normally well preserved and easily analysed in dried specimens. Using micro-computed tomography (μCT), we present the first wide geometric morphometric analysis on middle ear ossicles in mammals.

Eulipotyphla is an ideal group with which to test hypotheses relating to the functional role of the middle ear because it encompasses a wide range of ecological modes, covering a wide array of lifestyles from semi-aquatic, subterranean and semi-subterranean to terrestrial. The phylogenetic relationships among eulipotyphlans were recently clarified by molecular phylogenetic studies [33–42], providing us with the framework to test the evolutionary relationships between function and form. Both morphological and molecular evidence suggest that aquatic and subterranean specializations occurred multiple times independently among eulipotyphlans [37,43]. Our findings will aid ecological inferences based on fossil species, especially those of early mammalian forms (e.g. *Necrolestes*, *Ceratogaulus*) which are presumed to be subterranean animals [44,45].

## 2. Material and methods

### 2.1. Data collection

**Figure 1.**
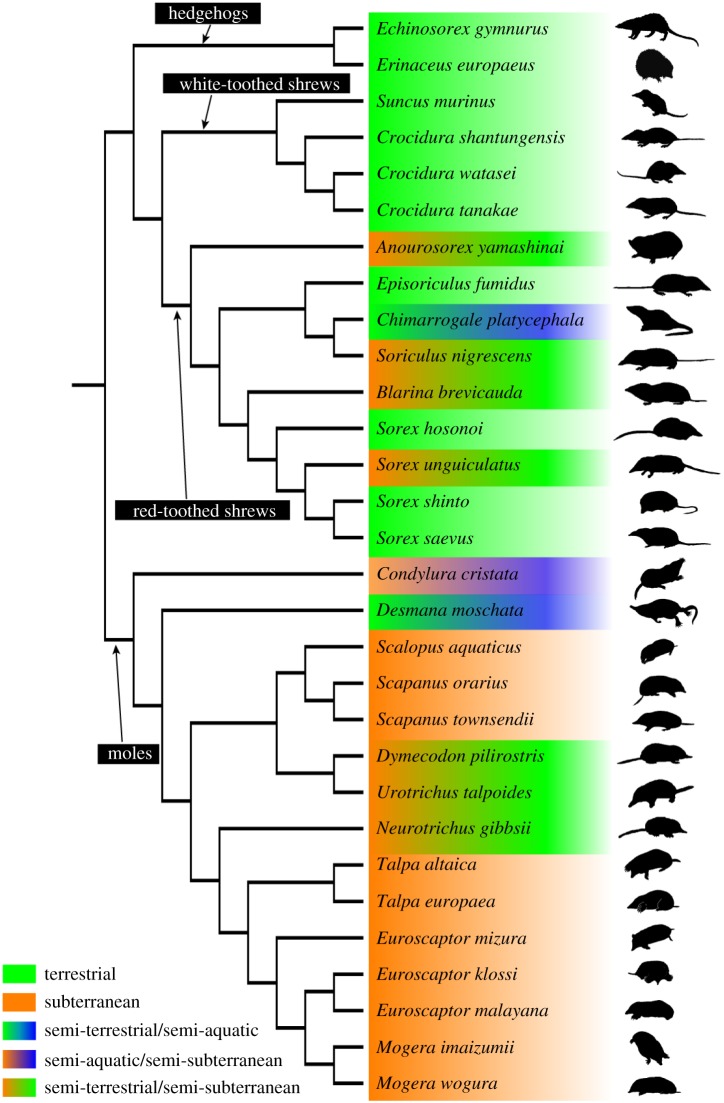
Phylogenetic tree based on molecular evidence and lifestyle of eulipotyphlans in this study.

We used 30 species of Eulipotyphla (figure 1), covering a wide array of lifestyles from semi-aquatic, subterranean, semi-subterranean to terrestrial. Nine species of red-toothed shrews (*Anourosorex yamashinai, Episoriculus fumidus, Chimarrogale platycephala, Soriculus nigrescens, Blarina brevicauda, Sorex hosonoi, Sorex unguiculatus, Sorex shinto and Sorex saevus*), four species of white-toothed shrews (*Suncus murinus, Crocidura shantungensis and Crocidura watasei* and *Crocidura tanakae*), two species of hedgehogs (*Echinosorex gymnurus and Erinaceus europaeus*) and 15 species of talpids (*Condylura cristata, Desmana moschata*, *Scalopus aquaticus, Scapanus orarius, Scapanus townsendii, Dymecodon pilirostris, Urotrichus talpoides, Neurotrichus gibbsii, Talpa altaica, Talpa europaea, Oreoscaptor mizura* (formerly *Euroscaptor mizura*, see [46] for its taxonomic status)*, Euroscaptor klossi, Euroscaptor malayana, Mogera imaizumii and Mogera wogura*) were used. The composite phylogenetic tree [40,47] and lifestyle of the studied species [48–68] are summarized in figure 1. The full-length (1140 base pairs (bp)) cytochrome *b* (cyt*b*) sequence data have been previously reported for all species [33–42], and between-species genetic distances of cyt*b* sequence were adopted as the phylogenetic framework of this study. Cyt*b* sequence was adopted because this is the only available sequence for all species investigated in this study. All specimens used in this study were considered to be adults because of full tooth eruption. These were stored at Kyoto University Museum (Kyoto, Japan), the University Museum of The University of Tokyo, the National Museum of Nature and Science (Tokyo, Japan) and the Smithsonian National Museum of Natural History (Washington DC, USA).

### 2.2. Micro-computed tomography scanning of specimens

We applied a μCT scanning technique to quantify the three-dimensional form and spatial arrangements of the ossicles. Serial cross-sectional images were obtained using a microfocal X-ray CT system at The University Museum, University of Tokyo (TX225-Actis, Tesco Corporation). Condylobasal lengths were measured on surface rendered reconstructions in Amira 5.2 (Visage Imaging, Berlin, Germany) for wet specimens to the nearest 0.01 mm. Mitutoyo digital calipers were used to measure condylobasal lengths for dry skulls to the nearest 0.1 mm. We conducted observations of the left-side temporal bone region for each specimen. In cases where the left side was destroyed, the right side was studied and mirror images constructed in Photoshop CS5 (Adobe Systems, San Jose, USA) were used. Scanning parameters were 95 or 100 kV tube voltage, 0.20 mA tube current and slice thickness and slice intervals were 12–34 µm. Cross-sectional images were reconstructed in a 512 × 512 matrix with a pixel size equivalent to the slice interval/thickness, producing isotropic voxels. Defining the threshold between the CT values of bone and non-bone regions by half maximum height method [69] using Amira 5.2, we segmented the bone boundaries of the middle ear ossicles with slice-by-slice manual adjustments (electronic supplementary material, table S1). The end-point of the anterior process of the malleus, which is attached to the ectotympanic, was determined by observing the slices from all three directions and checking the boundary between the ectotympanic and anterior process which was easily distinguishable by eye. The segmented surfaces and scanning details of the middle ears of studied specimens for which permission has been granted by owner museums are freely available at MorphoMuseum (http://dx.doi.org/10.18563/m3.3.2.e3) [70].

### 2.3. Geometric morphometric analysis and trait evolution analysis

**Figure 2.**
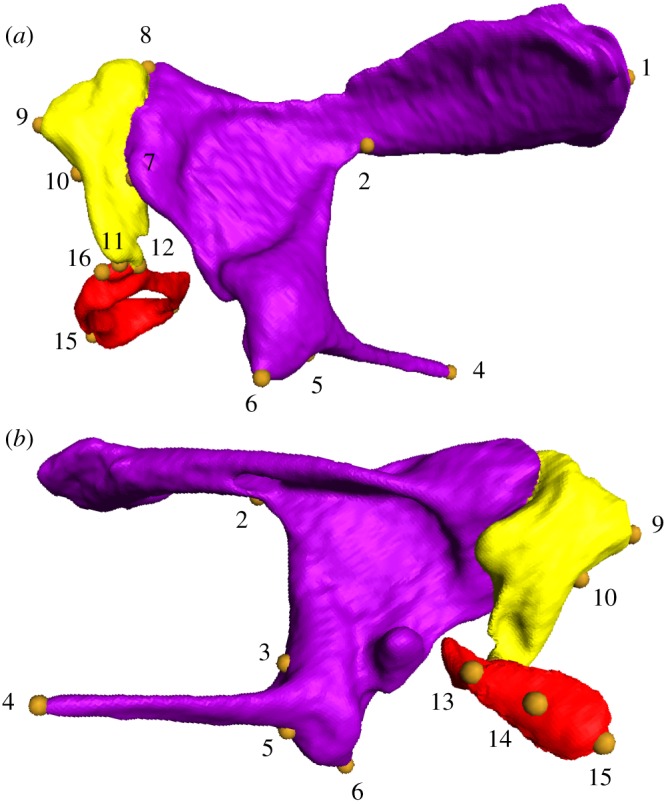
Landmarks taken in this study (ossicular example is of *Erinaceus europaeus*). (*a*) ventral view, (*b*) dorsal view of right-side.

After reconstruction, three-dimensional coordinates of 16 landmarks were collected in Amira 5.2 (figure 2). We performed a generalized procrustes analysis (GPA) [71] on the obtained landmark coordinate matrix of all samples. Using this GPA procedure, all configurations were translated to a common origin, then each specimen scaled with its centroid size [72] and rotated according to the least-squares criterion to minimize residual differences between configurations. GPA was performed using the MorphoJ program [73]. The obtained data set was then applied to principal component analysis (PCA) to explore the shape variation among all samples using the MorphoJ program.

Furthermore, we conducted a matrix correlation analysis to examine whether interspecific morphological distances more closely reflect ecology or phylogenetic history [74]. Morphological distances were computed from the procrustes distances of the GPA and summarized into a pairwise morphological distance matrix. Genetic distances were computed from full-length (1140 bp) cyt*b* sequence data of all previously reported species and obtained from GenBank [33–42], and then aligned by Clustal W [75]. Analyses were conducted using the maximum composite likelihood model [76]. The analysis involved 59 nucleotide sequences. The codon positions included were first, second, third and non-coding. All positions containing gaps and missing data were eliminated. A pairwise genetic distance matrix was computed from the number of base substitutions per site by averaging all the sequence pairs between groups using MEGA 6 [77]. Cty*b* sequences used in this study are available as electronic supplementary material. Using the ratios of daily behaviour should be the most appropriate approach to building a lifestyle distance matrix; however, such data were not available for most of the examined species. Therefore, according to reported lifestyle in the literature [43,48–54,60,78], we scored non-subterranean species as ‘0’, semi-subterranean species as ‘1’ and subterranean species as ‘2’ [79]. Then a lifestyle distance matrix was built from the score differences among species. Regarding lifestyle distances, we focused on the degree of fossoriality; the degree of aquatic lifestyle was not considered. *Chimarrogale platycephala* and *Desmana moschata,* which are semi-terrestrial/semi-aquatic animals and not subterranean, were scored as 0. *Condylura cristata*, a semi-aquatic but also a semi-subterranean species, was scored as 1.

Finally, the significance of the correlation between matrices was tested using Mantel's test [80], which randomly reorders the rows and columns of one matrix and recalculates the matrix correlation between the two matrices. The null hypothesis was that there would be no correlation between these two matrices. To determine the significance of the correlation, the randomized permutation was repeated 10 000 times. In addition to simple matrix correlation analyses, we conducted a partial Mantel's test between morphological distance matrix and ecological distance matrix, controlling against genetic distance matrix [74]. Both morphology and ecology may be affected by phylogenetic history, but partial Mantel's can compute the phylogeny-free correlation between morphological distance matrix and ecological distance matrix. All tests were processed using ZT software [74].

### 2.4. Impedance transform analysis

In addition to the above multivariate analysis, we conducted another comparison based on linear and area metrics of the ossicles to examine the auditory function of the ossicles. We quantified the impedance transformer ratio (ITR), which describes the ratio of the specific acoustic impedance at the oval window of the inner ear to the specific acoustic impedance at the tympanic membrane [81]. Lower ITRs indicate a greater effectiveness in transferring energy from the eardrum to the oval window [4]. Higher ITR values indicate reduced effectiveness in transferring sound energy, hence reduced hearing sensitivity [82,83]. Reduction of hearing sensitivity is argued to be essential to protect the inner ear from amplified acoustic pressure in underground tunnels, the so-called ‘stethoscope effect’ [83]. ITR has been a widely used measure to quantify the impedance matching of the mammalian middle ear [12,14–17,20,81], but it must be emphasized that the predictions made by ITR have been criticized for its methodological limitations (see the recent review by Mason [4] which highlights its technical issues). Although the anatomical measurements are appealingly simple, ITR does not take into account the frequency dependence of middle ear function and provides reasonable approximations only at low frequencies. We conducted this analysis to enable comparisons with previous studies which adopted this method, but we note that ITR values provided here should be treated as preliminary data that will contribute to our understanding of the auditory variation.

**Figure 3.**
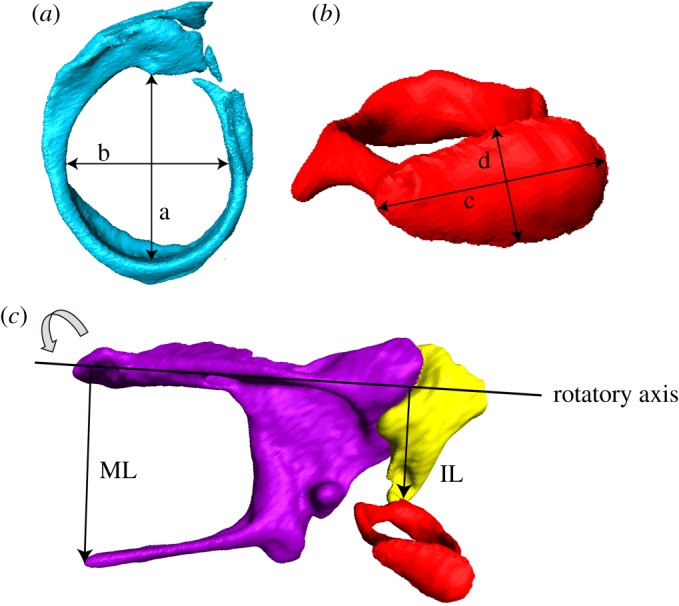
Measurements for impedance transformer ratios. (*a*) The longest axis of the tympanic membrane (a) and largest width perpendicular to the longest axis (b). (*b*) Footplate length (c) and footplate width (d) of the stapes. (*c*) Rotatory axis, ML and IL arms.

The ossicular anatomical terms used in this study are shown in figure 3. Tympanic membrane area (*A*_1_) was calculated as

$$A_{1}=\frac{1}{4}\pi ab,$$

in which *a* is the longest axis of the tympanic membrane and *b* is the largest width perpendicular to *a*. Similarly, the stapes footplate area (*A*_2_) was calculated as

$$A_{2}=\frac{1}{4}\pi cd,$$

where *c* is the longest axis of the stapes footplate and *d* is the largest width perpendicular to *c*. The area ratio (*A*_2_/*A*_1_) was then computed as the tympanic membrane area divided by the stapes footplate area [6].

The rotatory axis of the malleus and incus was defined as the line connecting the tip of the anterior process of the malleus to the tip of the short process of the incus [4]. The malleus lever (ML) arm was measured as the perpendicular distance from the tip of the manubrium to the rotatory axis, and the incus lever (IL) arm as that from the centre of the lenticular apophysis to the rotatory axis [4]. Then, the lever ratio (*B*) was calculated as

$$B=\frac{\text{ IL}}{\text{ML}}.$$

Following previous studies, the ITR of the middle ear was calculated as

$$\text{ITR }=\left(\frac{A_{2}}{A_{1}}\right)\times B^{2},$$

which describes the ratio of the specific acoustic impedance at the oval window to the specific acoustic impedance at the eardrum [81,84,85] (although the interpretations do not change, we note that this is the inverse of the conventional ITR values (e.g. [21,86]).

Box plots were used to show the median, minimum and maximum values of ITR for each lifestyle category, that is, semi-aquatic, terrestrial, semi-subterranean and subterranean. *Condylura cristata*, which is semi-aquatic and semi-subterranean, was assigned into both groups for boxplot comparisons. The Mann–Whitney *U*-test was conducted to compare the ITR among lifestyle groups. *Condylura cristata* was removed when comparing semi-subterranean and semi-aquatic species. Significance was determined after Bonferroni corrections. All statistical analysis were done in PAST v. 2.17c [87].

## 3. Results

### 3.1. Geometric morphometric analysis and trait evolution analysis

#### 3.1.1. Principle component analysis

**Figure 4.**
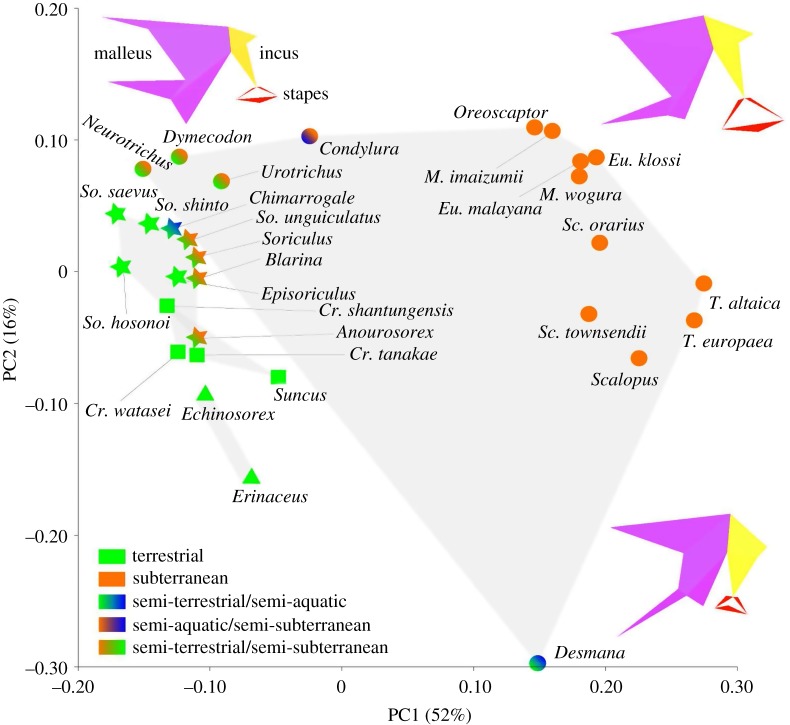
PCA scatter-plots and lifestyle representation of each species. Shapes of middle ear ossicles at axis extremes are given, the malleus in purple, incus in yellow and stapes in red. Talpids are shown in circles, red-toothed shrews in stars, white-toothed shrews in squares and hedgehogs in triangles. The grey shading represents the phylogenetic grouping of species, that is red-toothed shrews, white-toothed shrews, talpids and hedgehogs.

The PCA of the covariance matrix of the aligned procrustes coordinates for the 16 landmarks describing ossicle shape yielded 29 PCs. The basic statistics of the PCA are given in the electronic supplementary material, table S2. The first two PCs explained 68% of total shape variance, which substantially provided information of the total shape variation. Pearson's correlation between PC1 scores and condylobasal length was *r* = 0.43 (*p* = 0.02). Shape variation along PC1, which explained 52% of the whole variation, was particularly associated with anterior process length, manubrium orientation, stapes footplate area, size of incus and protrusion of orbicular apophysis (figure 4). The individuals with higher PC1 scores had a relatively shorter anterior process, enlarged stapes footplate, enlarged incus and less well-developed orbicular apophysis. In addition, they had a more rostrally, medially and ventrally oriented tip of the manubrium. The positive end of PC1 was dominated by highly subterranean talpids (figure 4). The whole clade of talpids (circles in figure 4) was distributed widely among PC1, but semi-subterranean talpids *Dymecodon pilirostris, Urotrichus talpoides* and *Neurotrichus gibbsii* had negative PC1 scores. The PC1 score of the semi-terrestrial and semi-aquatic *Desmana moschata* fell in the range of highly subterranean talpids. Red-toothed shrews (stars), white-toothed shrews (squares) and hedgehogs (triangles) all dominated the negative end of PC1, overlapping with semi-subterranean talpids (*Dymecodon pilirostris, Urotrichus talpoides* and *Neurotrichus gibbsii*). Within red-toothed shrews, semi-subterranean shrews (*Anourosorex yamashinai, Soriculus nigrescens*, *Blarina brevicauda* and *Sorex unguiculatus*) had higher PC1 scores than other terrestrial red-toothed shrews.

Pearson's correlation between PC2 scores and condylobasal length was *r* = −0.58 (*p* = 0.0002). The shape variation along the PC2, which explained 16% of the variation, was particularly related to length of the anterior process, length and orientation of the manubrium and size of incus. The individuals with higher PC2 scores possessed more medio-ventrally placed manubrium and latero-caudally placed, short, anterior processes (figure 4). Non-talpid eulipotyphlans, that is hedgehogs, red-toothed shrews and white-toothed shrews, were clearly separated by PC2 scores. Hedgehogs (*Echinosorex gymnurus and Erinaceus europaeus*) and *Desmana moschata* dominated the negative end of PC2. Talpids except *Desmana moschata* dominated the positive end of PC2.

#### 3.1.2. Mantel's and partial Mantel's test

Matrices of interspecific morphological distance, genetic distance and lifestyle distance are given in the electronic supplementary material, tables S3–S5. The results of the Mantel's and partial Mantel's tests are given in table 1. The Mantel's test on 28 species pairwise distance matrices revealed a very weak correlation between the morphological distance and genetic distance (*r* = 0.28, *p* = 0.003) and between lifestyle distance and genetic distance (*r* = 0.32, *p* = 0.0001). On the other hand, morphological distance showed a stronger correlation with lifestyle distance (*r* = 0.62, *p* = 0.0001). When genetic distance was controlled for in the partial Mantel's test, morphological distance and lifestyle distance were still strongly correlated (*r* = 0.59, *p* = 0.0001).

**Table 1.** Results of distance correlation analysis and *p*-values of Mantel's test and partial Mantel's test.

matrix comparisons	*r*	*p*
morphological distance versus lifestyle distance	0.62	0.0001
morphological distance versus genetic distance	0.28	0.003
lifestyle distance versus genetic distance	0.32	0.0001
morphological distance versus lifestyle distance (genetic distances controlled for)	0.59	0.0001

### 3.2. Impedance transform analysis

**Figure 5.**
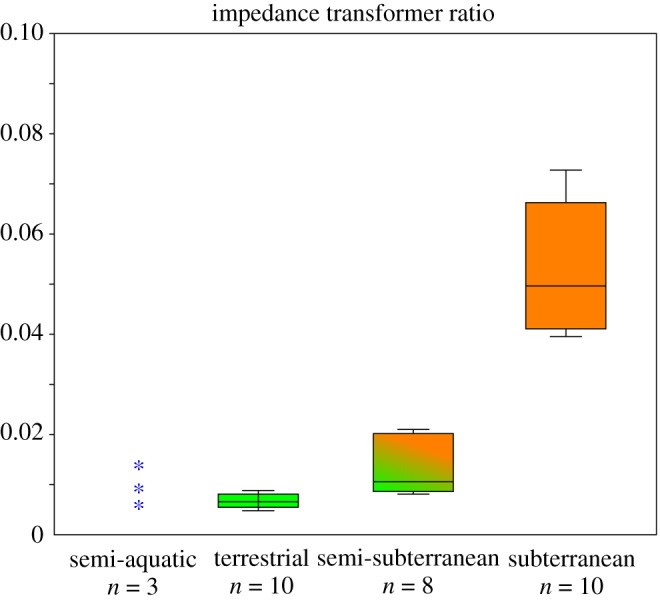
Boxplots of ITR for each lifestyle category. The 25–75% quartiles are drawn using a box. The median is shown with a horizontal line inside the box. The minimal and maximal values are shown with short horizontal lines. The semi-aquatic/semi-subterranean *Condylura cristata* was assigned into both semi-aquatic and semi-subterranean categories for this analysis.

The ML, IL, *a*, *b*, *c*, *d*, ITR and condylobasal length of all studied animals are given in the electronic supplementary material, table S6, and the ITR of each lifestyle category is summarized in figure 5. The ITR was significantly different between ecological groups: semi-aquatic versus semi-subterranean (*p* = 0.007), terrestrial versus semi-subterranean (*p* = 0.00001), semi-subterranean versus subterranean (*p* = 0.00001) and terrestrial versus subterranean (*p* = 0.00001) (electronic supplementary material, table S7). Terrestrial species showed the lowest and little variation of ITR. Subterranean was the highest and semi-subterranean was the intermediate of the two extremes. The ITR of semi-aquatic species was low, *Desmana moschata* (0.005) falling in the range of terrestrial species and *Chimarrogale platycephala* (0.014) being within the range of semi-subterranean species. The ITR of the semi-aquatic and semi-subterranean *Condylura cristata*, which was assigned into both semi-subterranean and semi-aquatic groups for this analysis, was 0.009. Among subterranean species, *Oreoscaptor mizura* (0.072) and *Scalopus aquaticus* (0.070) were the positive ends.

## 4. Discussion

### 4.1. Multivariate patterns and impedance transform function of the middle ear ossicles

Lack of strong correlation between morphological distance matrix and genetic distance matrix suggests that morphological variation of ossicular shape does not convey strong phylogenetic signals (table 1). On the other hand, significantly strong positive correlation was found between morphological distance matrix and ecological distance matrix even when the effect of phylogenetic inertia was statistically subtracted. These results indicate that the overall shape of the ossicles often converges according to lifestyle.

Highly subterranean talpids clustered in positive PC1 and terrestrial species clustered in negative PC1. Semi-subterranean species showed intermediate PC1 scores between the two extremes. These results suggest that the PC1 axis separates the studied animals according to the degree of fossoriality. The individuals with higher PC1 scores were characterized by relatively shorter anterior process, enlarged stapedial footplate, enlarged incus and less-developed orbicular apophysis. Quite strikingly, semi-subterranean talpids *Condylura cristata*, *Dymecodon pilirostris*, *Urotrichus talpoides and Neurotrichus gibbsii* [48,53,54,78] deviate from other truly subterranean talpids along PC1 and cluster with semi-subterranean shrews and terrestrial hedgehogs. Among red-toothed shrews (stars in figure 4), the semi-subterranean species *Anourosorex yamashinai, Blarina brevicauda*, *Soriculus nigrescens* and *Sorex unguiculatus* [25,44,51,55–57] dominate the high end of PC1 scores. This suggests that within soricids the middle ear ossicles of more subterranean species have independently shifted to more ‘talpid-like’ ossicles.

**Figure 6.**
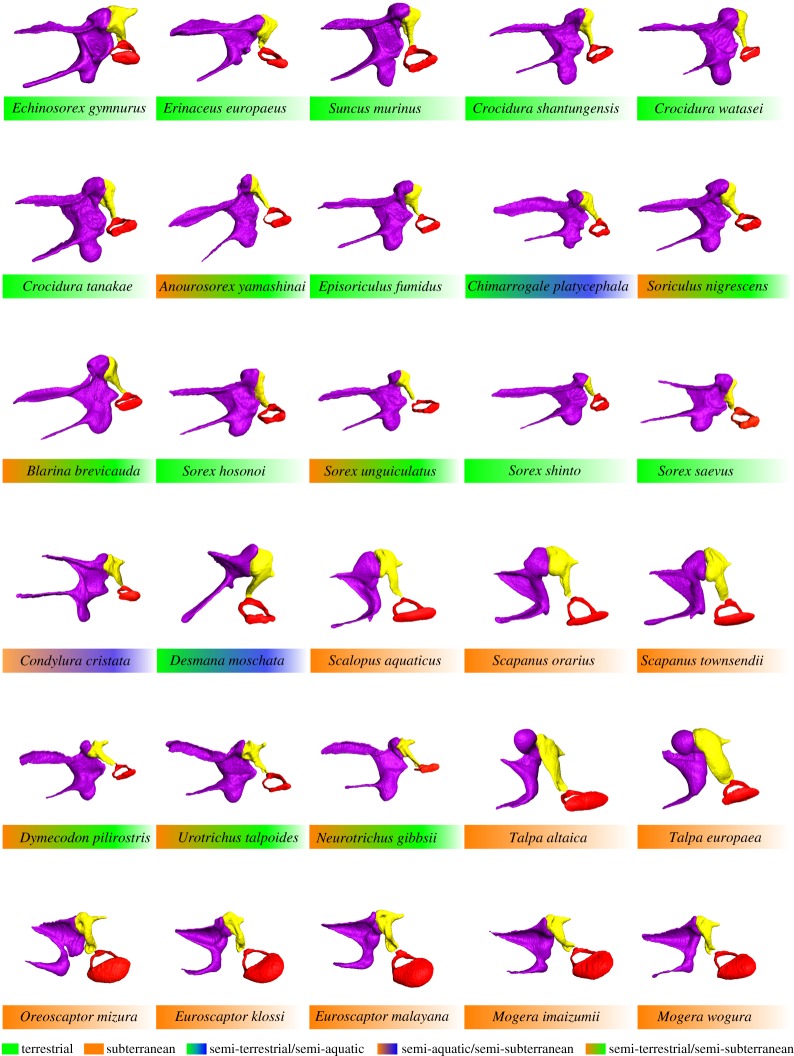
Reconstructed middle ear ossicles of studied species. Right middle ear ossicles are pictured from an internal view. The malleus is coloured in purple, incus in yellow and stapes in red.

PC2 mostly reflects allometric variation. Large-sized species, *Erinaceus europaeus*, *Echinosorex gymnurus* and *Desmana moschata,* showed lower PC2 scores. Along this axis, hedgehogs, red- and white-toothed shrews were well separated from one another, presumably due to similar body size within closely related species. Species with higher PC2 scores tend to possess more medio-ventrally placed manubrium, and latero-caudally placed short anterior process. *Desmana moschata*, which is located in the negative extreme of PC2, exhibit a remarkably slender and long manubrium. Orientation of the manubrium is distinctive compared to other animals, making the angle between the manubrium and anterior process nearly vertical, whereas in most-studied species the orientation of the manubrium and anterior process is nearly parallel (figure 6).

It would be most advantageous for the airborne hearing of fossorial mammals to be tuned towards low frequencies, which propagate better than high frequencies in the underground environment [11,83]. The hearing of terrestrial hedgehogs and shrews is reported to be restricted to sonic range and high frequency [13,88], whereas that of talpids is mainly restricted to low-frequency sounds. *Talpa europaea* and *Mogera robusta*, both highly subterranean, are reported to respond to frequencies from 0.01 to 22 kHz and to be most sensitive around 3 kHz [13]. Further studies on actual hearing capabilities are needed, but our findings that subterranean talpids are equipped with an enlarged incus and stapes footplate are overall consistent with previous studies on other subterranean rodents, marsupials and golden moles [12,14,16,18,20] and what has been found previously in several talpid species [15,16].

Impedance transformer analysis indicated that ITR, which was calculated with the inverse values of area ratio and lever ratio, was significantly higher in subterranean species compared with species of other lifestyles (figure 5). ITR of the semi-subterranean group was intermediate between subterranean and terrestrial ones. Among the genus *Sorex*, semi-subterranean *Sorex unguiculatus* exhibits a higher ITR than the terrestrial congeners, such as *Sorex hosonoi*, *Sorex shinto* and *Sorex saevus*. The high ITR in *Sorex unguiculatus* is achieved by both increased footplate/tympanic membrane area ratio and increased IL/ML ratio. *Sorex unguiculatus*, a shrew distributed around Hokkaido and the east coast of Russia, is reported to spend 33% of the day underground and construct tunnel systems up to approximately 30 cm in depth [55–57]. *Blarina brevicauda*, a sister taxon to *Sorex,* exhibits the highest ITR among the semi-subterranean group and shows an even higher ITR than semi-subterranean talpids such as *Condylura cristata*, *Dymecodon pilirostris, Urotrichus talpoides* and *Neurotrichus gibbsii*. *Blarina brevicauda* is not exclusively subterranean but is known as the most subterranean American shrew [60]. Although ITR successfully discriminated groups of differing ecological niches in our study (figure 5), as noted earlier ITR values presented here should be treated with caution as preliminary information. The middle ear function is affected by many different factors, ITR values presenting one among many others [89].

### 4.2. Variation in malleus morphology

**Figure 7.**
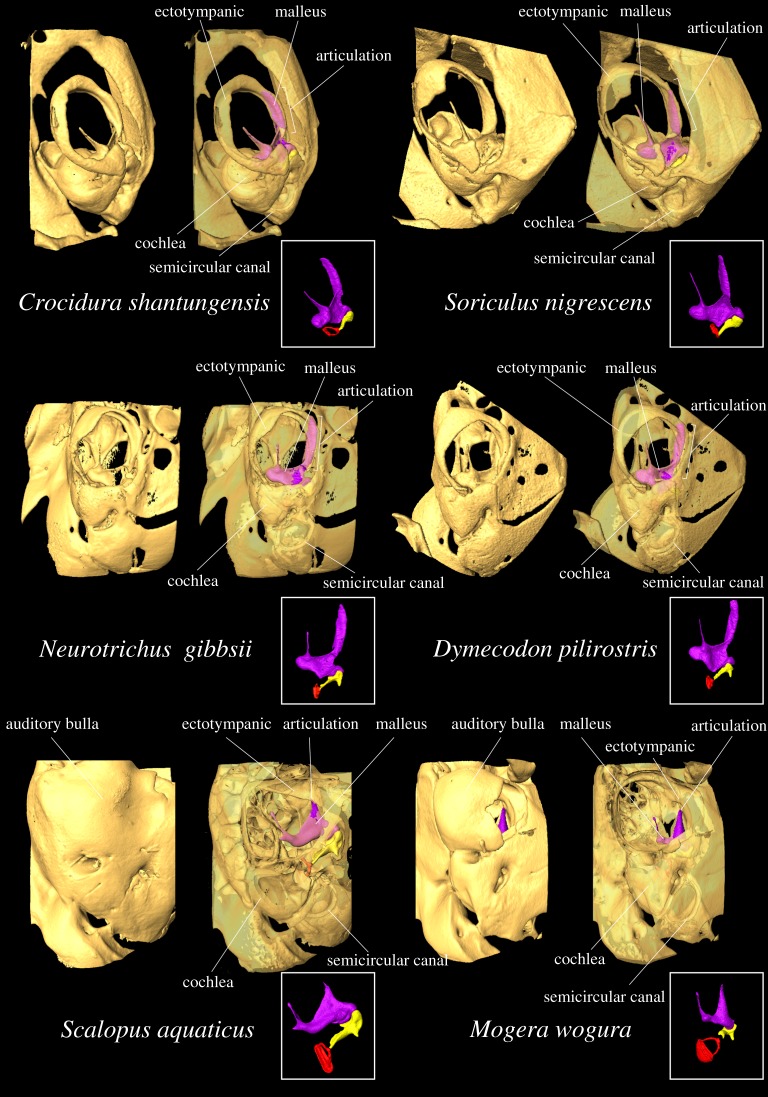
Reconstructed μCT images of the auditory region of selected species showing the articulations between the malleus and ectotympanic. Images are of left side auditory region. For each species, non-transparent surface rendering, transparent rendering and the middle ear ossicles are shown. Both non-transparent and transparent images are given to show the outer and inner morphology of the ear region, respectively. Skulls in the upper row are those of terrestrial species, middle are those of semi-subterranean species and lower are those of highly subterranean species. Note the slight articulation between the tip of the anterior process and ectotympanic in the highly subterranean *Scalopus aquaticus* (lower left) and *Mogera wogura* (lower right), whereas the articulation is broad in terrestrial species (upper row) and semi-subterranean (middle row). *Mogera wogura* and *Neurotrichus gibbsii* are closely related, *Scalopus aquaticus* and *Dymecodon pilirostris* are closely related, respectively (see the electronic supplementary material, figures S1–S6 for close-up images).

More subterranean species were characterized by a relatively shorter anterior process and less-developed orbicular apophysis. A shorter anterior process leads to a reduction in the attachment area to the ectotympanic compared with longer anterior processes. This allows loose and more flexible articulation between the anterior process and ectotympanic, which is a pattern found in subterranean rodents [15,16]. We observed that the anterior process of more terrestrial eulipotyphlans is attached firmly to the ectotympanic, whereas more subterranean eulipotyphlans tend to show slight and loose connections (figure 7; electronic supplementary material, figures S1–S6). A low-stiffness articulation between ossicles and skull should improve low-frequency sound transmission at low frequencies [90]. Such articulation is advantageous because airborne low-frequency sound can travel further than high-frequency sound in the underground environment [11].

Although a hypertrophied orbicular apophysis is peculiar to white-toothed shrews [14,16,91] (figure 6, electronic supplementary material, table S9), it is known that such an orbicular apophysis is not observed in fully subterranean moles [14]. The lack of orbicular apophysis is reported in subterranean rodents (e.g. *Cratogeomys castanops*, *Cryptomys hottentotus*, *Fukomys mechowi*, *Geomys bursarius*, *Georychus capensis* and *Spalax ehrenbergi*) and the subterranean marsupial mole (*Notoryctes typhlops*) [2,16,91]. Among talpids, the presence of a well-developed orbicular apophysis is reported in two semi-subterranean talpids (*Condylura cristata* and *Urotrichus talpoides*) [92]. According to our study, all shrews and hedgehogs and semi-subterranean talpids (*Condylura cristata, Dymecodon pilirostris*, *Urotrichus talpoides* and *Neurotrichus gibbsii*,) are equipped with a well-developed hypertrophied orbicular apophysis, while this is lacking or rudimentary in all other talpids (figure 6; electronic supplementary material, table S9; see also [70]). An orbicular apophysis is slightly present in the aquatic *Desmana moschata*. These results are in agreement with a previous report by Mason [15] which found prominent orbicular apophyses in semi-subterranean *Neurotrichus gibbsii* and *Parascalops breweri*. The orbicular apophysis is relatively reduced in semi-subterranean shrews compared with terrestrial shrews (figure 6). The subterranean golden moles also lack an orbicular apophysis [19]. A reduced orbicular apophysis, which theoretically brings the ossicular centre of mass closer to the anatomical axis, appears to be a signal of subterranean specialization [19,93]. Bringing the centre of mass of the ossicles closer to the anatomical axis allows reducing transmission of bone-conducted vibrations to the inner ear by the ossicular inertial route [15,94]. Reducing transmission of bone-conducted vibration leads to inhibition of the low-frequency noise which disturbs the subterranean species in hearing airborne sounds [15,16]. While an orbicular apophysis is commonly found among mammals, a more developed orbicular apophysis is found in micromammals such as bats and rodents [2]. A larger orbicular apophysis theoretically shifts the centre of mass away from the anatomical axis, allowing small-sized mammals to increase the moment of inertia of the malleus and incus without increasing absolute ossicular mass [93,95]. Increasing the moment of inertia of the malleus and incus and simultaneously increasing articular stiffness of the ossicles enhances the sharpness of tuning [95].

### 4.3. Evolutionary patterns of aquatic adaptation

The inclusion of aquatic species in this study allowed us to explore the patterns of aquatic adaptations. While the degree of subterranean lifestyle appears to be well reflected in morphological variation, no clear pattern was found for aquatic specialization (figures 4 and 5). Our samples included three semi-aquatic species, *Chimarrogale platycephala, Condylura cristata* and *Desmana moschata,* all of which live around fresh water and hunt prey mostly in water [52,54,96]. Neither convergent clustering in PC scores nor proportionally enlarged ossicles were confirmed (electronic supplementary material, table S2). The ITR of *Chimarrogale platycephala, Condylura cristata* and *Desmana moschata* fall in the range of terrestrial and semi-subterranean species (figure 5; electronic supplementary material, table S6). The angle between the manubrium and anterior process in *Desmana moschata* is nearly vertical, which results in elongation of the ML. This increases the in-lever versus the out-lever ratio of the middle ear ossicles and contributes to a decreased ITR. However, while the ITR of semi-aquatic *Desmana moschata* is distinctively low among talpids, it does not exceed the range of terrestrial species. Possible explanations for the lack of a clear pattern in the middle ear are that these animals may use alternative strategies for hearing, such as bone conduction as in pinnipeds [6,97].

### 4.4. Evolutionary patterns of subterranean adaptation

The morphology of the auditory region in eulipotyphlan mammals clearly reflects a specialization for subterranean habits and confirms convergent evolution in multiple lineages. The middle ears of burrowing shrews have acquired a more ‘talpid-type’ morphology, deviating from terrestrial shrews and hedgehogs. Among talpids, both morphological and molecular evidence supports that their common ancestor was not fully specialized for underground lifestyle and that further specialization for subterranean lifestyle occurred independently in the Eurasian lineage (*Talpa altaica, Talpa europaea, Oreoscaptor mizura, Euroscaptor klossi*, *Euroscaptor malayana*, *Mogera imaizumii* and *Mogera wogura*) and the American lineage (*Scalopus aquaticus, Scapanus orarius* and *Scapanus townsendii*) [37,43,98]. If that is the case, our study indicates that whereas semi-subterranean talpids (*Condylura cristata*, *Dymecodon pilirostris, Urotrichus talpoides* and *Neurotrichus gibbsii*) retained non-specialized middle ear ossicles, subterranean Eurasian and American lineage became convergent in terms of the functional aspect of the middle ear.

Our geometric morphometric analyses incorporating genetic distances demonstrate that the overall shape of the ossicles in eulipotyphlan mammals often converges according to lifestyle and that middle ear morphology in eulipotyphlans has been able to evolve flexibly with ecological shifts. Although no clear pattern was found for aquatic specialization, we confirmed that subterranean adaptation should include a relatively shorter anterior process of the malleus, enlarged incus and enlarged stapes footplate. Reduction of the orbicular apophysis was also found in more subterranean species. These morphological settings improve sound transmission at low frequencies and reduce transmission of bone-conducted vibrations. This study builds a base for further investigations on ossicular variation among mammals. We assume that the traits summarized here can greatly contribute to detect subterranean adaptations in extinct taxa.

## Supplementary Material

Electronic_supplementary_data_cytb_sequence

## Supplementary Material

Electronic_supplementary_figures

## Supplementary Material

Electronic_supplementary_tables

## Data accessibility

All raw measurements and landmark data are provided in the electronic supplementary material. The surface data of studied specimens for which permission has been granted by owner museums is available from MorphoMuseum. http://dx.doi.org/10.18563/m3.3.2.e3.
