# Supplementary material for: Into the dark: patterns of middle ear adaptations in subterranean eulipotyphlan mammals
Source: R Soc Open Sci. 2017 Sep 20;4(9):170608. doi: 10.1098/rsos.170608 (PMC5627103; doi:10.1098/rsos.170608)
Supplement: Electronic_supplementary_figures [file rsos170608supp2.docx]

**Supplementary Figures**

**for**

***Into the dark: patterns of middle ear adaptations in subterranean eulipotyphlan mammals***

Daisuke Koyabu, Misato Hosojima, Hideki Endo

*Correspondance: koyabu@um.u-tokyo.ac.jp

This supplementary material contains the images of auditory regions of selected specimens, showing the articulation between the malleus and ectotympanic.

**Supplementary Figure S1.** Close-up image of the auditory region of *Crocidura shantungensis*.

**Supplementary Figure S2.** Close-up image of the auditory region of *Soriculus nigrescens*.

**Supplementary Figure S3.** Close-up image of the auditory region of *Neurotrichus gibbsii.*

**Supplementary Figure S4.** Close-up image of the auditory region of *Dymecodon pilirostris.*

**Supplementary Figure S5.** Close-up image of the auditory region of *Scalopus aquaticus.*

**Supplementary Figure S6.** Close-up image of the auditory region of *Mogera wogura.*


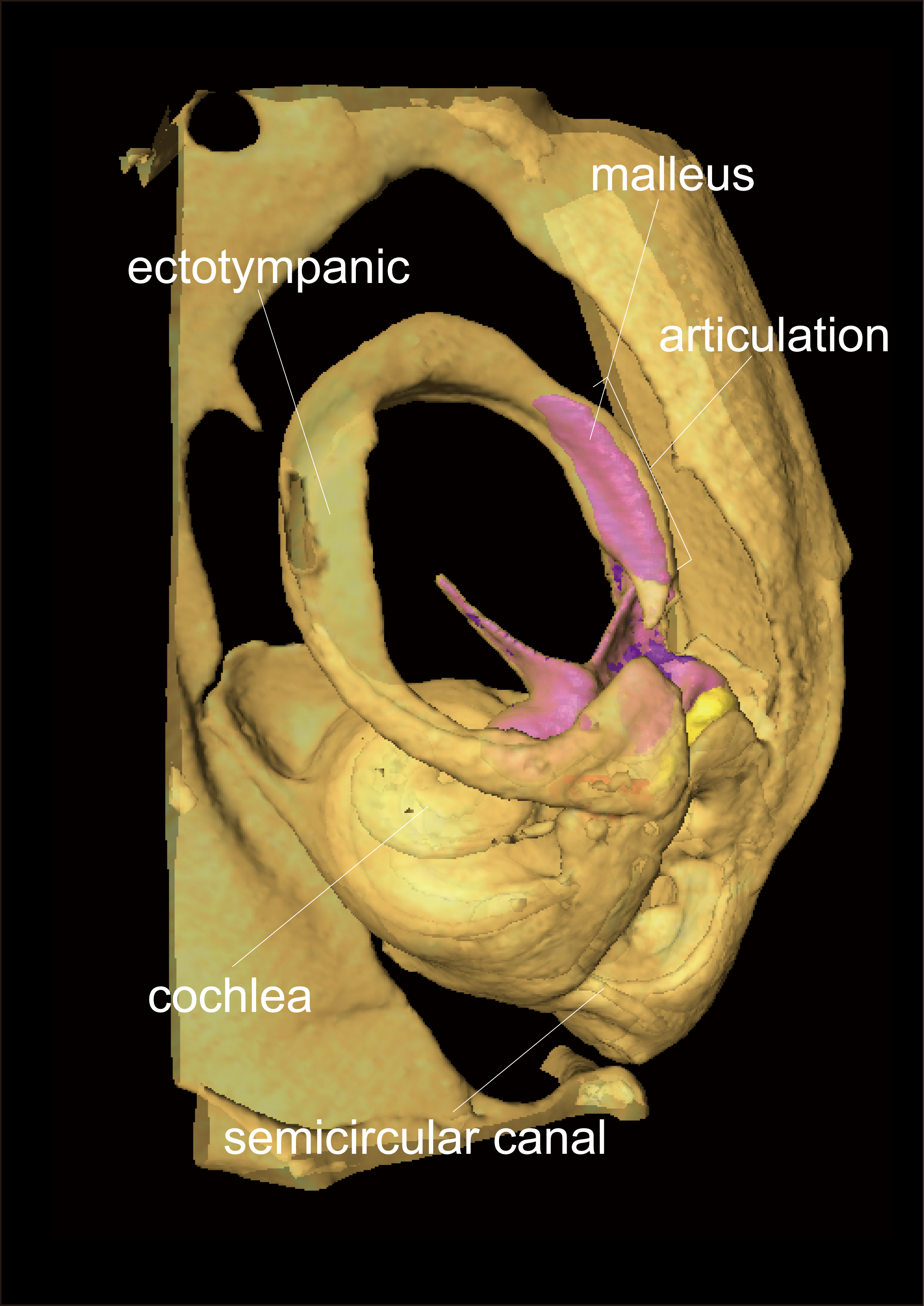


Figure S1. Close-up image of the auditory region of *Crocidura shantungensis.*


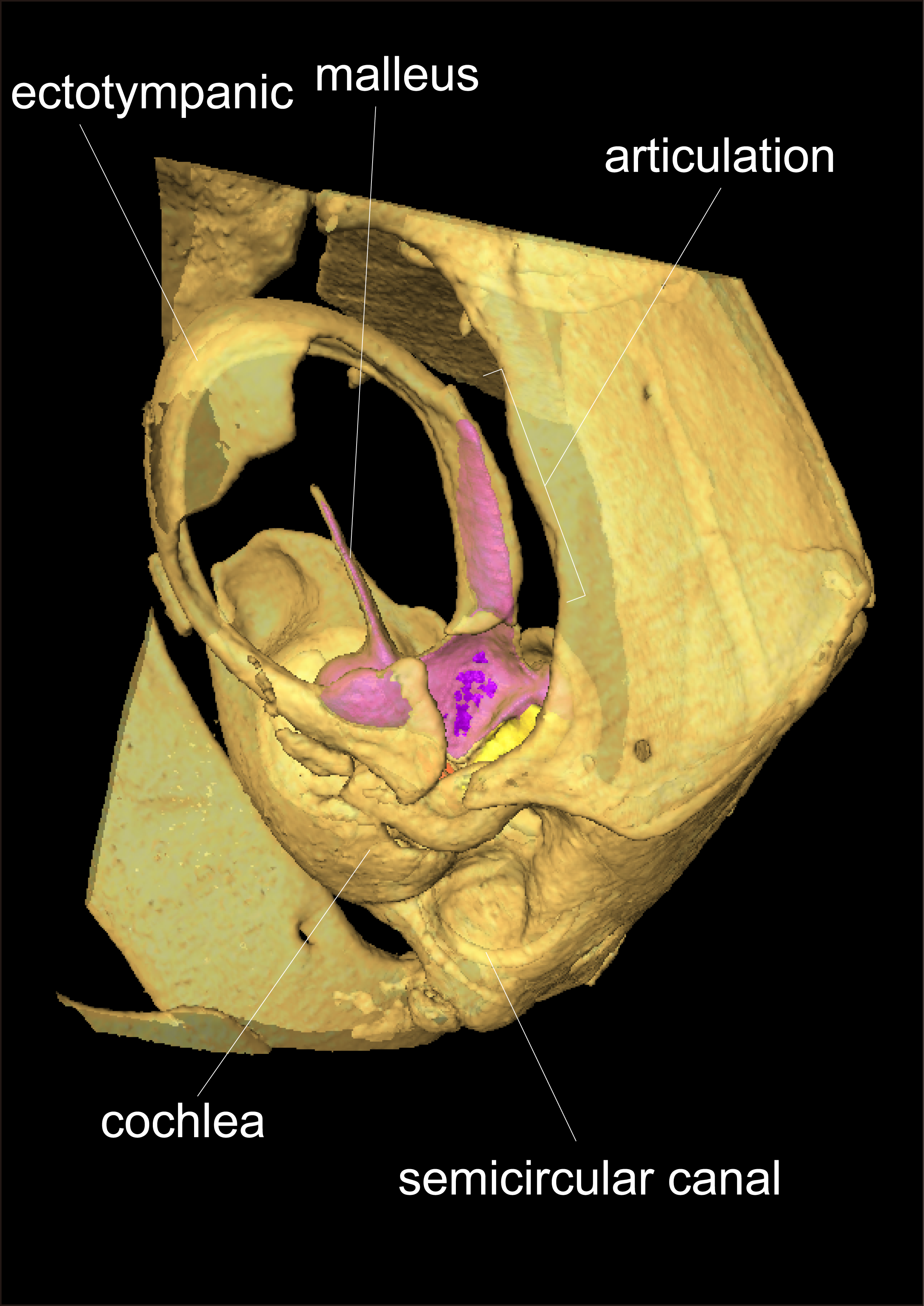


Figure S2. Close-up image of the auditory region of *Soriculus nigrescens.*


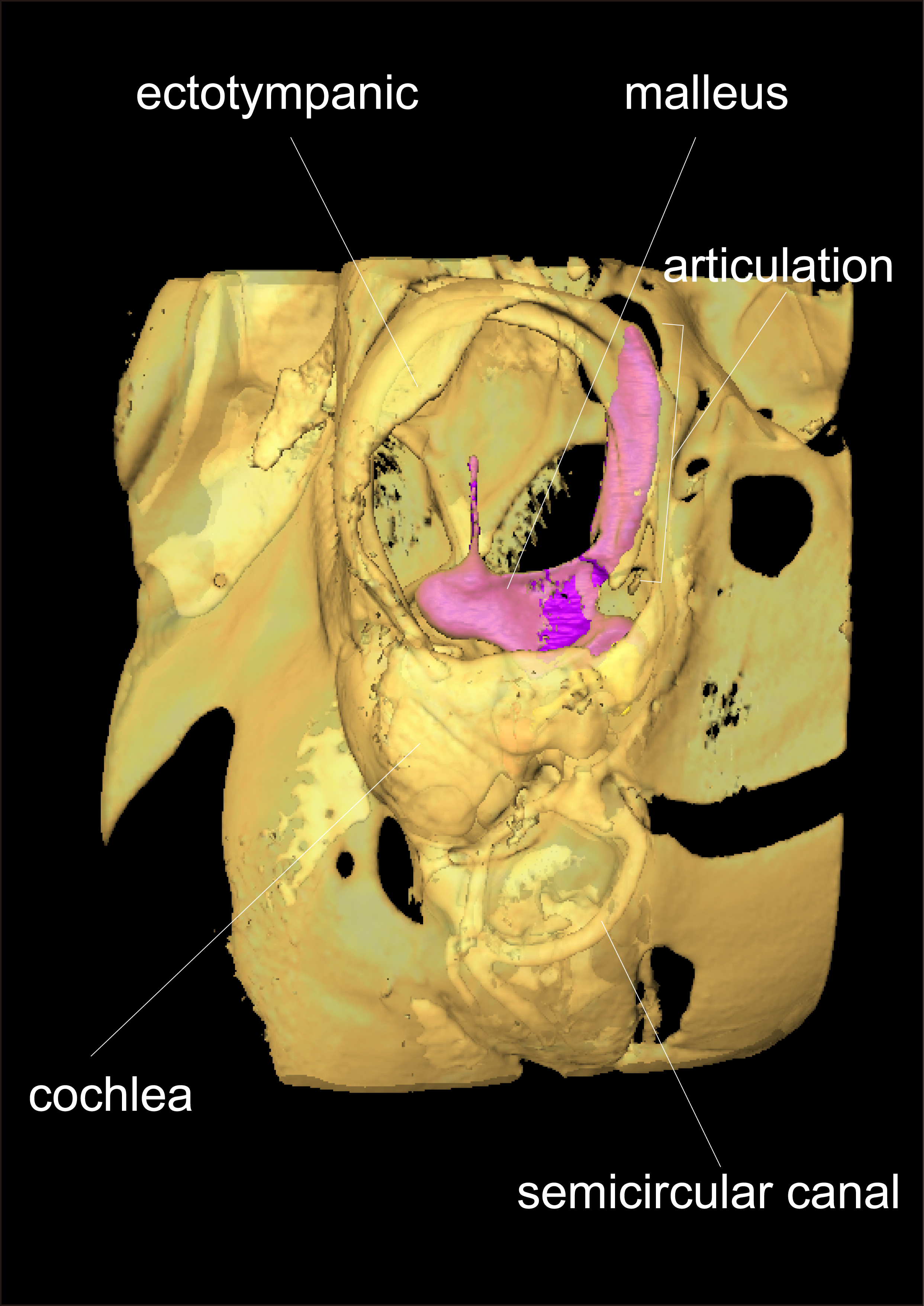


Figure S3. Close-up image of the auditory region of *Neurotrichus gibbsii.*


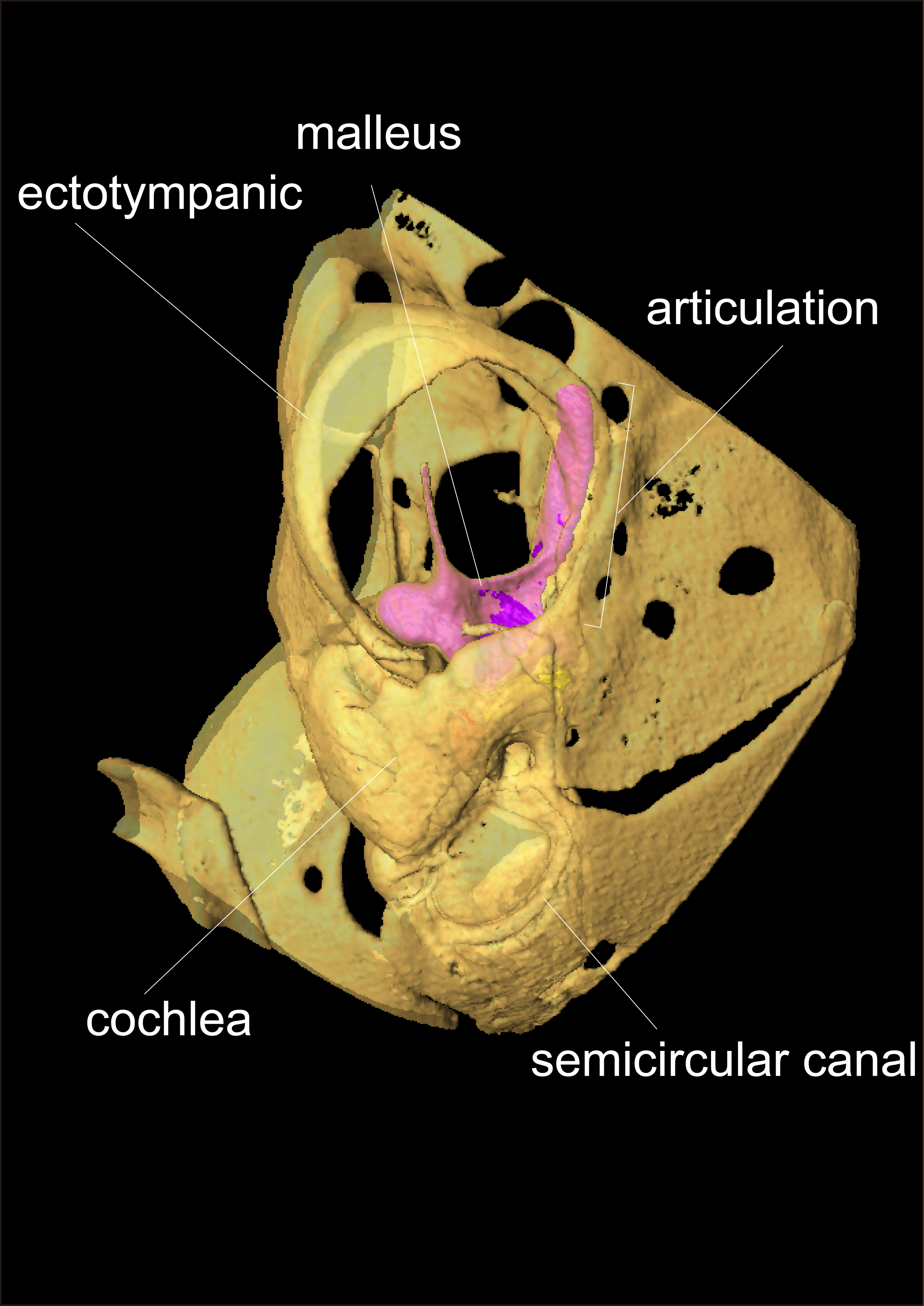
Figure S4. Close-up image of the auditory region of *Dymecodon pilirostris.*


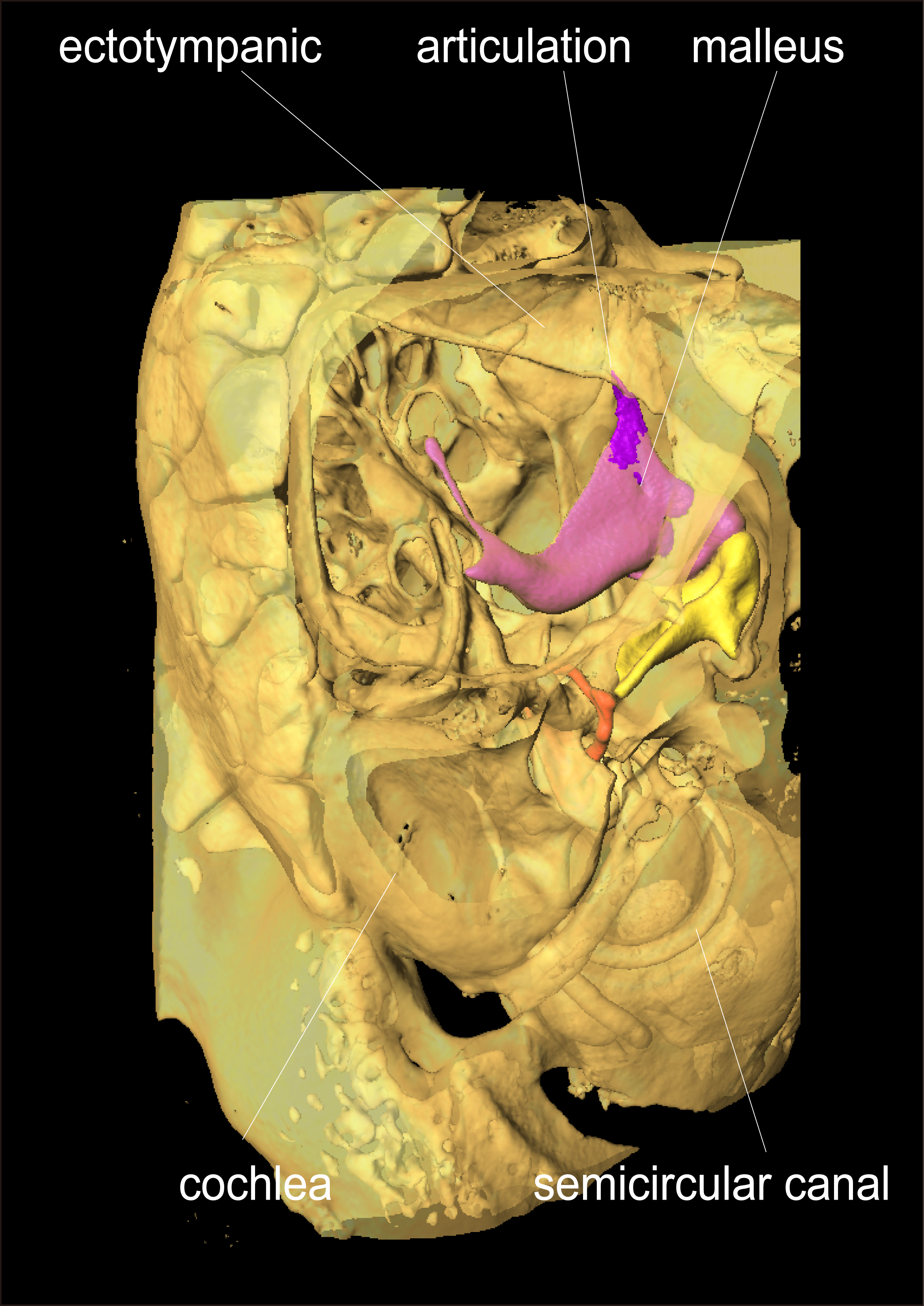


Figure S5. Close-up image of the auditory region of *Scalopus aquaticus.*


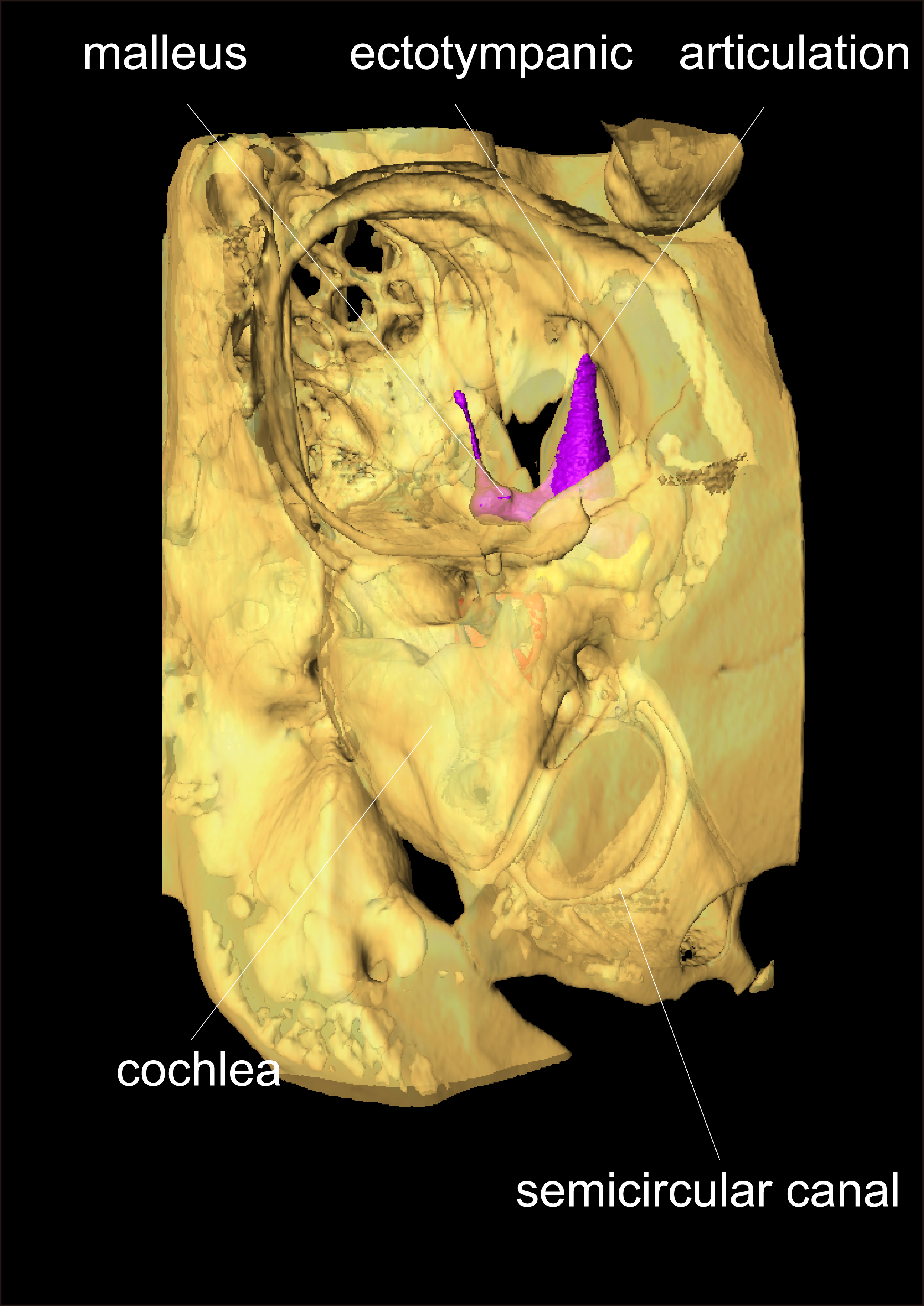


Figure S6. Close-up image of the auditory region of *Mogera wogura.*
